# Double Common Bile Duct Associated With Choledochal Cyst: A Rare Developmental Anomaly

**DOI:** 10.7759/cureus.91079

**Published:** 2025-08-26

**Authors:** Muhammad Yahya Khan, Abdul Rehman, Muzamil Aslam Chaudhary

**Affiliations:** 1 General Surgery, Children Hospital Faisalabad, Faisalabad, PAK; 2 Pediatric Surgery, Children Hospital Faisalabad, Faisalabad, PAK; 3 Emergency, St. Luke's General Hospital, Kilkenny, IRL; 4 Emergency Medicine, Royal Albert Edward Infirmary, Wigan, GBR

**Keywords:** choledochal cyst excision, double common bile duct (dcbd), hepaticojejunostomy, magnetic resonance cholangiopancreatography (mrcp), vomiting pediatric

## Abstract

Duplication of the extrahepatic bile duct is a highly uncommon congenital variation of biliary anatomy and may occur in conjunction with other biliary or pancreatic abnormalities. We describe the case of a 3-year-old girl who presented with abdominal discomfort and vomiting. Magnetic resonance cholangiopancreatography (MRCP) revealed a cystic lesion consistent with a choledochal cyst (CC). Intraoperatively, a double common bile duct (DCBD), an atypical anomaly characterized by duplication of the extrahepatic bile duct, was identified. Treatment involved unifying the duplicated ducts into a single channel, followed by a Roux-en-Y hepaticojejunostomy and cholecystectomy. The patient recovered well postoperatively and was advised to undergo long-term monitoring and follow-up due to potential late complications. This case highlights the importance of anticipating and recognizing rare biliary anomalies such as DCBD, as unawareness may increase the risk of intraoperative injury and underscores the need for lifelong surveillance.

## Introduction

During early biliary development, the extrahepatic bile duct arises from the hepatic diverticulum and undergoes a transient epithelial occlusion followed by recanalization; aberrations in this process can produce duplication of the bile duct. Choledochal cysts (CCs) are rare congenital dilatations of the biliary tree, typically presenting in infancy or early childhood. Recent genomic studies have implicated chromosomal anomalies, notably duplications and microdeletions at 17q12 encompassing the HNF1B gene, in the development of CCs. HNF1B is a transcription factor essential for bile duct development, and its dosage alterations have been linked to biliary anomalies, including CCs [1]. Incidence varies geographically - approximately 1 in 1,000 live births in parts of East Asia versus 1 in 100,000-150,000 in Western countries [2, 3].

An extremely rare and often under-reported anomaly is the duplication of the extrahepatic bile duct, referred to as a double common bile duct (DCBD). First noted by Vesalius in the 16th century and subsequently detailed by several authors [4, 5]. Several classification systems have been proposed, with Choi et al.'s modification offering a more detailed framework based on ductal configuration and drainage pattern [6]. DCBD may occur in isolation but has been reported to coexist with other hepatobiliary anomalies, including CCs, pancreaticobiliary maljunctions, and occasionally gastrointestinal malignancies, thereby complicating diagnosis and management. The presence of DCBD can be missed preoperatively, on magnetic resonance cholangiopancreatography (MRCP), and has important consequences: it increases the risk of inadvertent bile duct injury, incomplete cyst excision, misplacement of the biliary-enteric anastomosis, and postoperative leak or stricture. Long-term follow-up should be planned, given the potential for late complications.

## Case presentation

A 3-year-old girl, previously healthy, presented to our outpatient pediatric surgical clinic with a two-month history of intermittent abdominal pain and non-bilious vomiting. Parents also reported a recent weight loss of 4 pounds and a decrease in her oral intake over the last three months, but did not report any jaundice or fever. She had no past medical or surgical history.

Physical examination revealed a 4.5 x 3.5 cm soft, ill-defined, non-tender cystic mass palpable in the right upper abdomen, extending from the right hypochondrium to the umbilicus. Her laboratory investigations revealed deranged liver function tests and raised bilirubin levels (Table 1).

**Table 1:** Laboratory investigations WBC: white blood cell; AST: aspartate aminotransferase; ALT: alanine transaminase; MCV: mean corpuscular volume

Test	Result	Normal range
WBC count	14.3 × 10^9 ^/L	5-17 × 10^9 ^/L
Hemoglobin	12.3 g/dL	11.5-14 g/dL
Platelets	539,000 × 10^9 ^/L	150,000-400,000 × 10^9 ^/L
MCV	87.4 fl	83-101 fl
AST	147 U/L	15-50 U/L
ALT	138 U/L	5-55 U/L
Total Bilirubin	9.12 mg/dL	<1.5 mg/dL
Direct Bilirubin	5.92 mg/dL	<0.2 mg/dL
Indirect Bilirubin	3.2 mg/dL	<1.3 mg/dL

**Figure 1:**
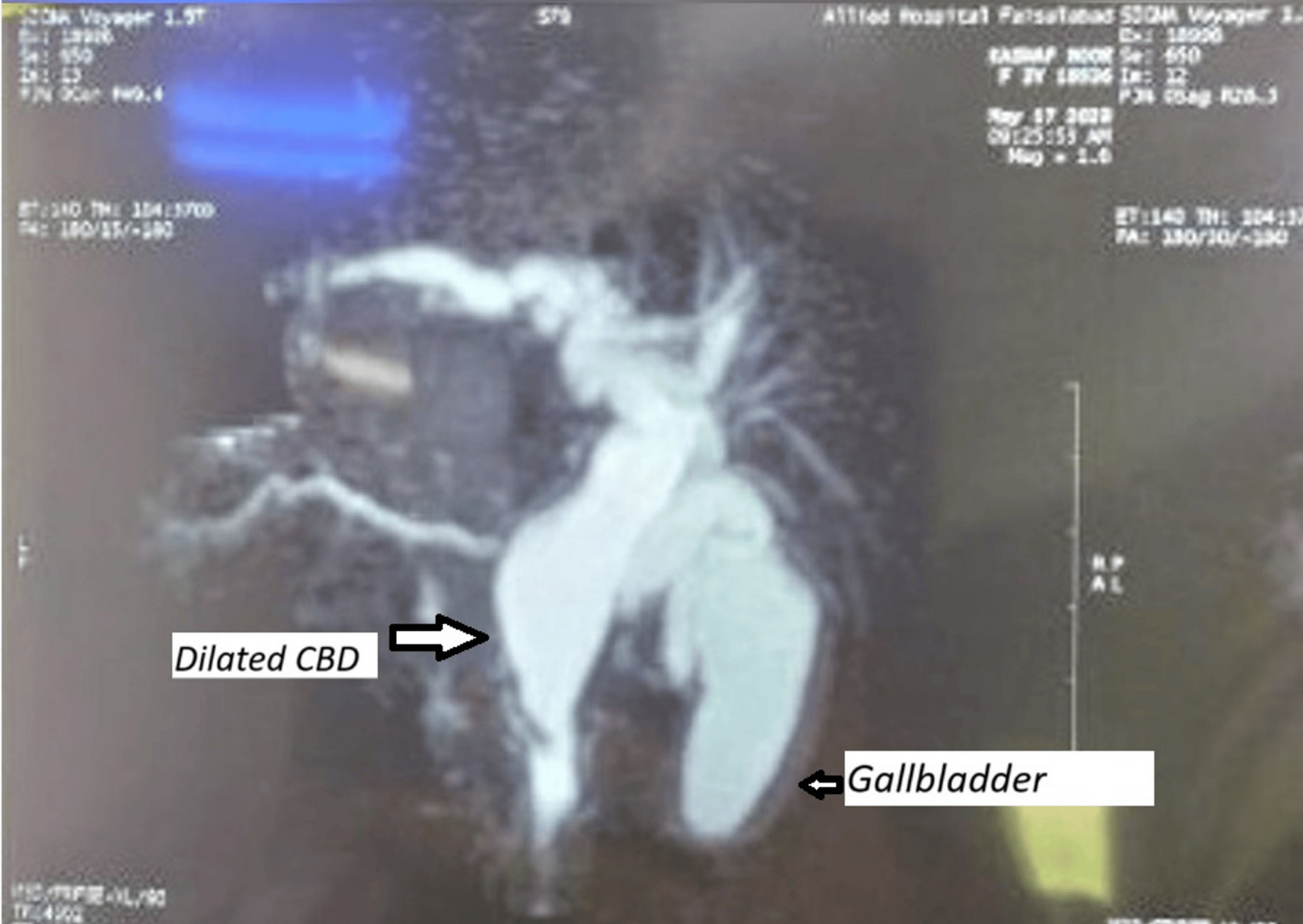
MRCP showing dilated CBD and gallbladder MRCP: Magnetic resonance cholangiopancreatography, CBD: Common bile duct

An abdominal ultrasound demonstrated a 4 cm cystic dilatation of the common bile duct and mild dilation of the intrahepatic ducts. Further evaluation with magnetic resonance cholangiopancreatography (MRCP) confirmed fusiform dilatation of the intrahepatic and extrahepatic bile ducts, suggestive of a Type IV choledochal cyst as per the Todani classification (Figure 1). No ductal duplication was identified preoperatively.

**Figure 2:**
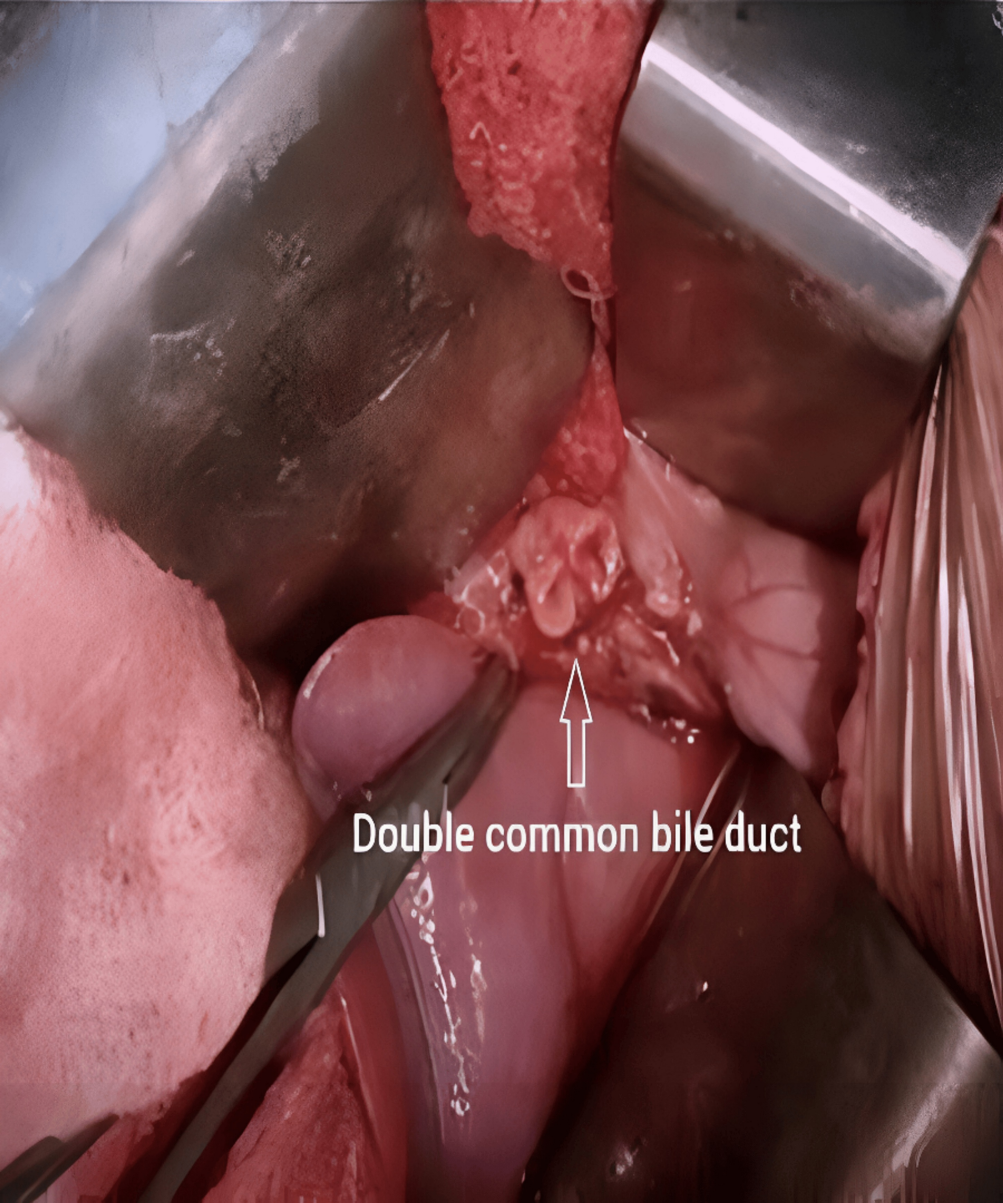
Intraoperative picture of DCBD DCBD: Double Common Bile Duct

**Figure 3:**
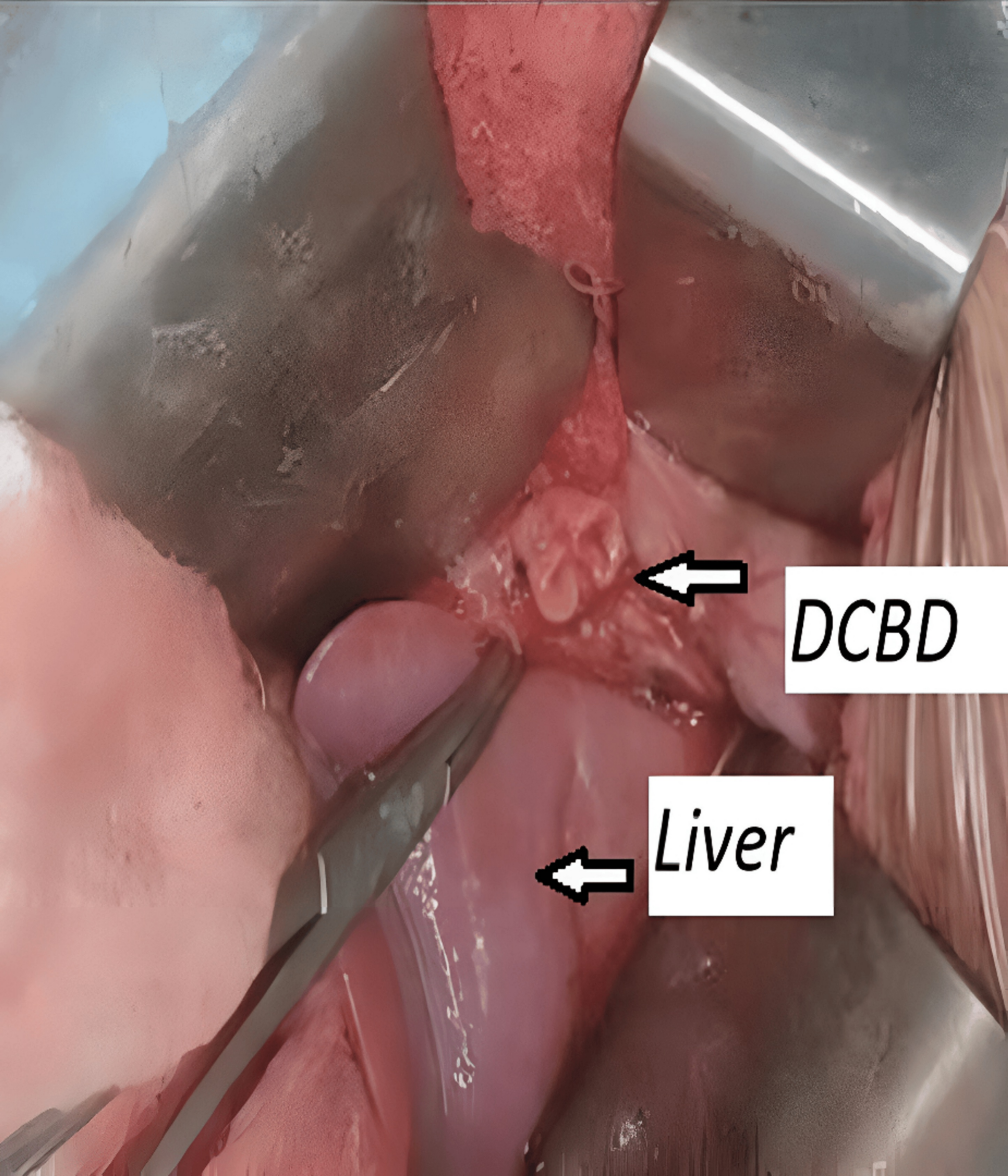
Intraoperative picture of DCBD and liver DCBD: Double Common Bile Duct

The patient was taken for elective open excision of a choledochal cyst through a right subcostal incision. Intraoperatively, an unanticipated double common bile duct (DCBD) was identified, characterized by a thin intraluminal septum producing complete duplication up to the confluence of the right and left hepatic ducts. This anomaly had not been visualized on preoperative MRCP (Figures 2, 3).

The interluminal septum was excised to unify the lumens, thereby converting the duplicated duct into a single channel. The choledochal cyst and gallbladder were then completely excised following meticulous dissection from the adjacent portal vein and hepatic artery. Biliary-enteric continuity was restored with a Roux-en-Y hepaticojejunostomy, in which a Roux limb of jejunum was mobilized to the hepatic hilum and anastomosed end-to-side to the common hepatic duct using PDS 5-0 suture. Intestinal continuity was re-established via side-to-side jejunojejunostomy, and a subhepatic drain was placed.

The operative duration was approximately 4 hours, with an estimated blood loss of less than 150 mL. No intraoperative cholangiogram was performed. Postoperatively, the patient was managed with intravenous fluids, antibiotics, and analgesia, with early initiation of ambulation and gradual advancement of diet. Recovery was uneventful, with no evidence of bile leakage or hemorrhage. The drain was removed prior to discharge on postoperative day 7. Histopathologic analysis confirmed a double-lumen common bile duct separated by a thin membranous septum without dysplasia or malignancy. At the two-week follow-up, the patient demonstrated satisfactory oral intake, absence of vomiting, and overall clinical improvement. Long-term surveillance in the surgical clinic was arranged.

## Discussion

The simultaneous presence of a choledochal cyst (CC) and a double common bile duct (DCBD) represents a highly unusual anatomical and clinical scenario, with fewer than 20 cases reported in the literature to date. While CCs are more frequently seen in pediatric populations, especially in East Asia, the global incidence remains low, estimated at 1 in 100,000 to 150,000 live births in Western countries [1]. The pathogenesis of CC remains incompletely understood. Two predominant hypotheses have been proposed: pancreaticobiliary maljunction (PBM) and congenital bile duct stenosis [1]. PBM is a developmental anomaly in which the pancreatic and bile ducts unite outside the duodenal wall, resulting in an abnormally long common channel. In this setting, the sphincter of Oddi is unable to effectively control the pancreaticobiliary junction, permitting bidirectional reflux. The mixing of bile and pancreatic secretions can activate pancreatic enzymes, which in turn injure the bile duct epithelium, provoke inflammation, and ultimately contribute to cystic dilatation characteristic of CC. Nevertheless, PBM is demonstrable in only about 50-80% of CC cases. An alternative explanation is congenital stenosis of the bile duct, which has been linked to a relative paucity of ganglion cells and neurons in the common bile duct wall. This abnormal innervation may cause uncoordinated contractility and elevated intraluminal pressure proximally, leading to progressive cystic dilatation [7].

**Figure 4:**
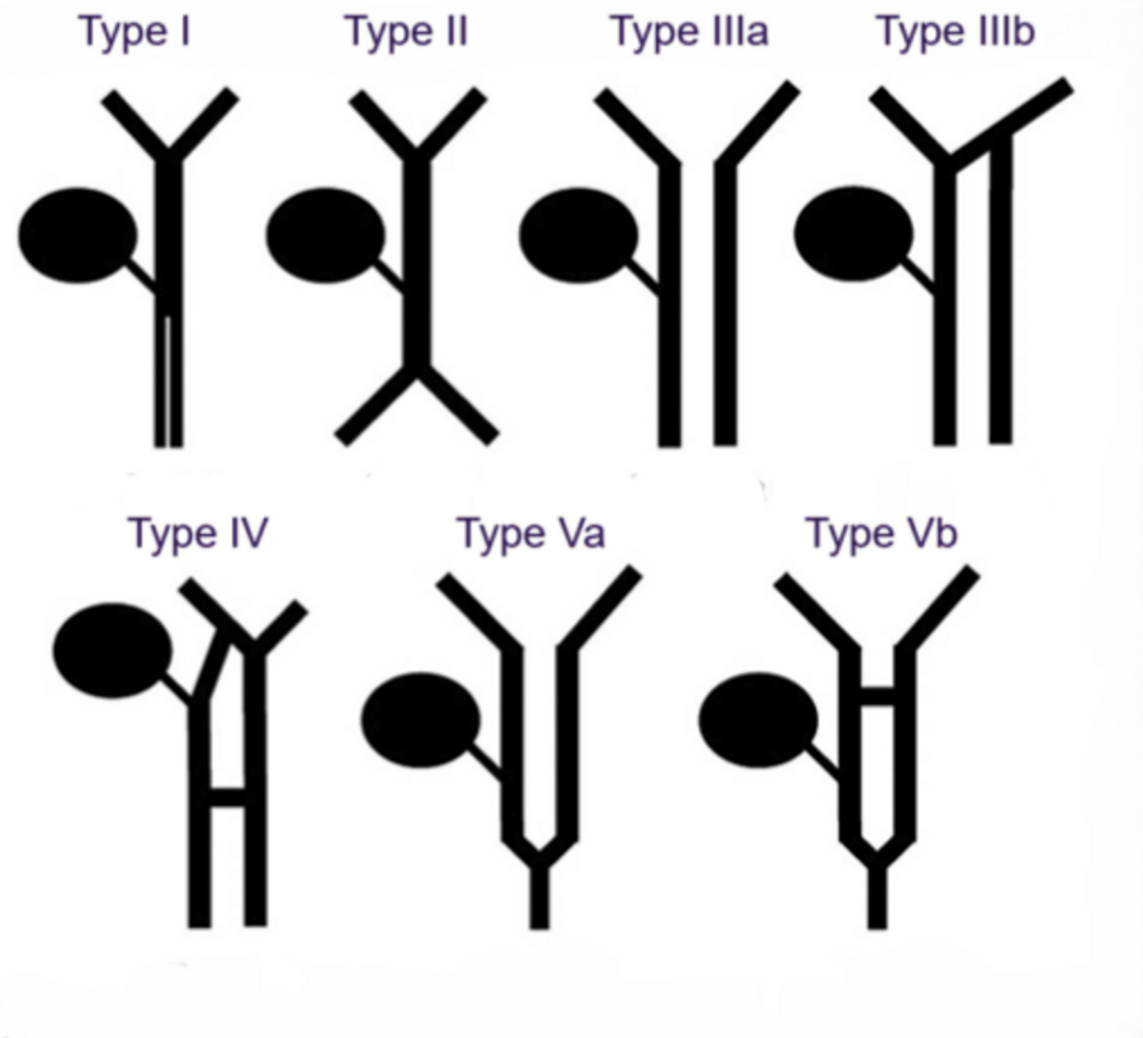
DCBD classification proposed by Choi et al. Type I: septum separating CBD; Type II: CBD bifurcates to drain separately; Type III: double biliary drainage without (a) or with (b) intrahepatic communicating channels; Type IV: double biliary drainage with extrahepatic communicating channels; Type V: single biliary drainage of DCBDs without (Va) or with (Vb) communicating channels. DCBD: double common bile duct; CBD: common bile duct Image credits: Created by authors

**Figure 5:**
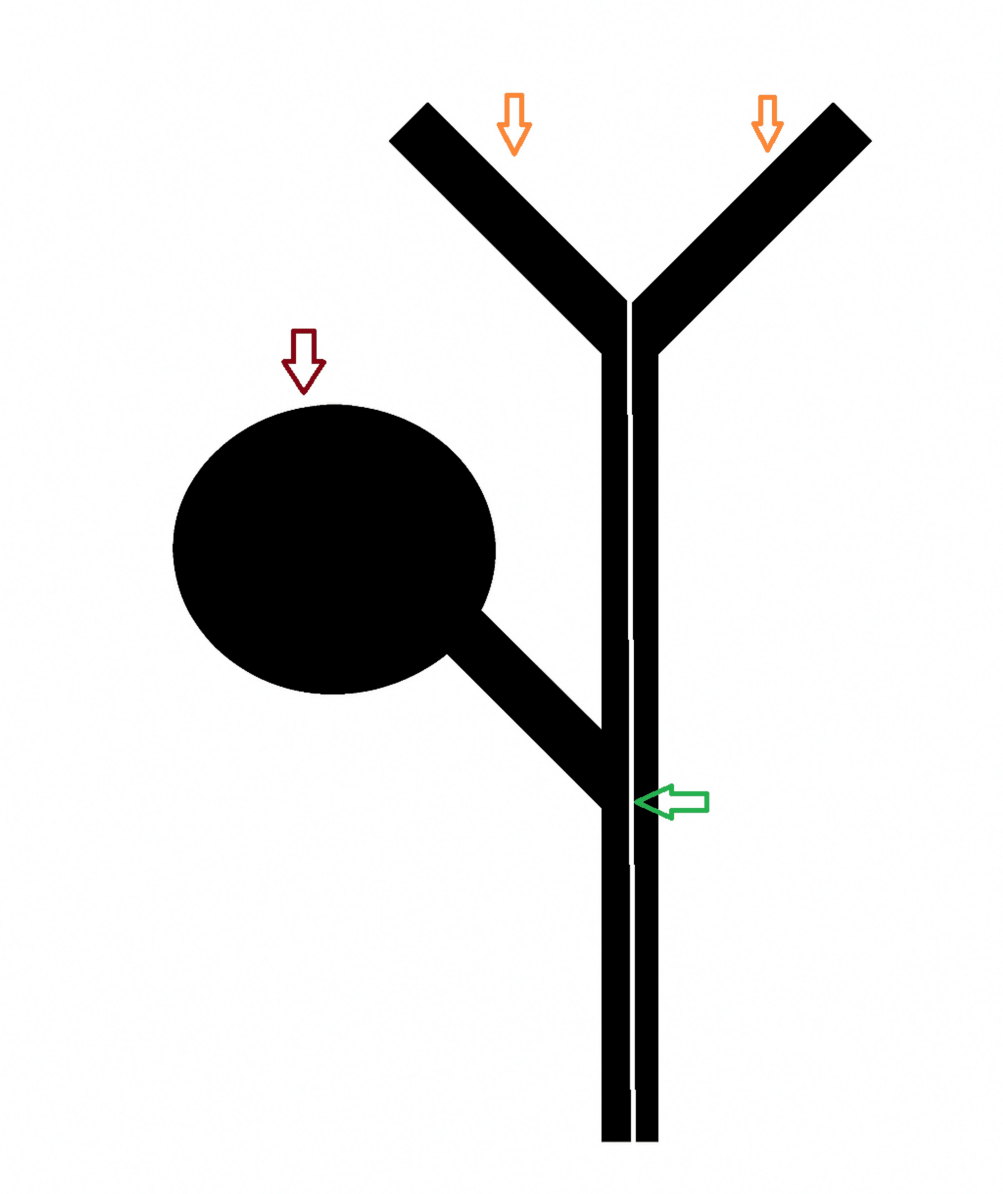
Diagram of our DCBD variant, showing gallbladder (red arrow) and the interluminal membranous septum (green arrows) in the CBD extending up to the junction of the left and right hepatic ducts (orange arrows). DCBD: double common bile duct; CBD: common bile duct Image credits: Created by authors

On the other hand, DCBD is a much rarer congenital anomaly, first described in anatomical literature by Vesalius in 1543 [2]. A review of the Japanese and Chinese literature revealed that CC and DCBD co-existed in 10.6 and 33.3% of the reported cases of DCBD, respectively [5]. It may remain undetected until surgery due to its rarity, atypical anatomy, and the limitations of imaging studies. The latest classification system of the DCBD was proposed by Choi et al. based on morphology, which did not take into account the aberrant common bile duct exits [6]. They described five subtypes, which involved seven variants (Figure 4). Since then, many new variants of double common bile duct have been reported. A case report by Sheng et al. demonstrated the various forms DCBD can take and emphasized that many variants cannot be classified under the existing systems [8]. Our case further substantiates this observation, as the identified DCBD variant in our patient did not conform to the variants proposed by Choi et al., reinforcing the need for an updated and expanded classification framework (Figure 5).

Unlike CC, preoperative diagnosis of DCBD is challenging, with most cases diagnosed surprisingly during surgery or after inadvertent biliary tree injury. Imaging modalities such as MRCP are considered highly sensitive for biliary tree evaluation; however, they may still fail to detect certain anatomical duplications, particularly in pediatric patients, where small duct size, a very thin membranous septum (as in our case), and the absence of pronounced dilation may obscure visualization [9, 10]. In addition, high-quality MRCP images are challenging to obtain in children due to the small caliber and short length of the pediatric bile ducts, as well as limited sedation and motion artifacts [11]. This limitation is consistent with prior findings from Nievelstein et al., who reported challenges in diagnosing biliary anomalies using standard imaging techniques in children [12].

The recommended treatment for choledochal cyst is complete cyst excision due to the continued risk of malignancy as well as other complications such as pancreatitis, cholangitis, and ductal stenosis. Clinically, both CC and DCBD have independent and additive risk of malignancy, particularly cholangiocarcinoma, if left untreated or if partially excised [13]. The risk is attributed to chronic biliary stasis, recurrent infections, and epithelial dysplasia. A meta-analysis conducted by Ten Hove et al. found that out of the 2904 cases of CC, 11% cases developed malignancy. The prognosis of those who develop a malignancy is poor, with a 5-year survival rate of only 5% [14]. Granata et al. illustrated the radiologic patterns of malignancy in choledochal cysts and emphasized the importance of early diagnosis and complete excision [13].

Surgically, Roux-en-Y hepaticojejunostomy with cyst excision remains the gold standard for CC treatment [2]. The excision of the CC provides excellent results with an 89% event-free rate and an overall 5-year survival rate above 90% [15, 16]. Draining the CC without complete resection could be harmful because it carries a very high risk of malignant transformation [13]. In cases complicated by ductal anomalies like DCBD, intraoperative vigilance is crucial. In our case, after dividing the septum of the DCBD and converting it into a single-lumen bile duct, we preferred a single anastomosis with the jejunum over a double anastomosis because of the theoretical advantages of reduced tissue damage and fewer postoperative anastomotic complications. The operating surgeon should be prepared to encounter and adapt to the unanticipated anatomical variations, despite thorough preoperative imaging. While MRCP remains an invaluable tool in planning for CC excision, it may not always delineate intricate anomalies, such as aberrant ductal openings or accessory ducts [8, 17]. Choi et al. showed that anatomical variations in the biliary tract can increase surgical complexity and the risk of injury if unanticipated [6]. Our case echoes this, as the DCBD went undetected until surgery, necessitating immediate intraoperative adaptation.

Surgical removal of choledochal cysts in children is typically well tolerated [18]. In the immediate postoperative period, complications such as anastomotic leakage, hemorrhage, surgical site infection, acute pancreatitis, or the development of biliary or pancreatic fistulas can occur. Reported rates of early issues like wound infections vary between 0% and 17% [19]. Long-term problems can include anastomotic narrowing, intrahepatic stones, liver cirrhosis, cholangitis, and even malignant transformation. Compared with adults, children are less likely (10-25%) to develop benign anastomotic strictures associated with recurrent cholangitis [19]. In our case, no immediate complications were identified, and regular follow-ups have been arranged to monitor for possible late sequelae.

Finally, patients with CC remain at high risk of developing metachronous cholangiocarcinomas, even after complete resection, and lifelong follow-up is advised. However, there is no agreement on what constitutes an acceptable follow-up strategy [13]. Surveillance typically includes periodic imaging and liver function monitoring. A systematic review by Jonathan et al. proposed that for patients treated primarily for cyst types I and IV and unresected type V should have annual liver function tests, carbohydrate antigen 19-9 (Ca 19-9) measurement, and biannual ultrasound assessment for 20 years post cyst resection, with biannual liver function testing, Ca 19-9 measurement, and three-yearly ultrasound assessment thereafter [20].

## Conclusions

This case underscores the critical importance of anticipating and recognizing rare anatomical variations, such as DCBD, during pediatric hepatobiliary surgery. Despite the use of advanced preoperative imaging modalities like MRCP, such anomalies may remain undetected until encountered intraoperatively, posing diagnostic and surgical challenges. A vigilant surgical approach - characterized by a high index of suspicion, meticulous dissection, and flexibility in operative strategy - is essential to minimize the risk of iatrogenic injury and ensure optimal outcomes. Beyond reinforcing the need for surgical caution, this case contributes to the existing literature by highlighting a previously underreported variant of DCBD, thereby supporting the call for a more comprehensive classification system that encompasses newly identified anomalies. Furthermore, given the established association between choledochal cysts and malignancy, our experience emphasizes the importance of long-term surveillance even after complete cyst excision and biliary reconstruction. These insights may guide future research aimed at refining diagnostic strategies and developing evidence-based follow-up protocols for patients with rare biliary anomalies.
